# The potential of hexatungstotellurate(VI) to induce a significant entropic gain during protein crystallization

**DOI:** 10.1107/S2052252517012349

**Published:** 2017-10-27

**Authors:** Christian Molitor, Aleksandar Bijelic, Annette Rompel

**Affiliations:** a Universität Wien, Fakultät für Chemie, Institut für Biophysikalische Chemie, Althanstrasse 14, Wien 1090, Austria

**Keywords:** polyoxotungstate, crystallization additives, crystal contacts, liquid–liquid phase separation, solvent entropy

## Abstract

The limiting factor in protein crystallography is still the production of high-quality crystals. In this regard, the authors have recently introduced hexatungstotellurate(VI) (TEW) as a new crystallization additive, which proved to be successful within the liquid–liquid phase separation (LLPS) zone. Presented here are comparative crystal structure analyses revealing that protein–TEW binding not only induces and stabilizes crystal contacts, but also exhibits a significant impact on the solvent-driven crystallization entropy, which is the driving force for the crystallization process. Upon the formation of TEW-mediated protein–protein contacts, the release of water molecules from the hydration shells of both molecules, *i.e.* TEW and the protein, causes a reduced solvent-accessible surface area, leading to a significant gain in solvent entropy. Based on the crystal structures of aurone synthase (in the presence and absence of TEW), insights have also been provided into the formation of a metastable LLPS, which is caused by the formation of protein clusters, representing an ideal starting point in protein crystallization. The results strongly encourage the classification of TEW as a valuable crystallization additive.

## Abstract 2

Hexatungstotellurate(VI) (TEW), an Anderson–Evans-type polyoxometalate (POM), was found to be a promising cocrystallization agent that mediates new strong crystal contacts and contributes significantly to the solvent entropy which presumably leads to a lower free energy of protein crystallization. The results presented herein strongly encourage the use of TEW as a powerful additive in protein crystallization, particularly within or near the liquid–liquid phase separation (LLPS) region.

## 1. Introduction   

X-ray crystallography is currently the primary method for the structure determination of proteins, which is also reflected by the content of the Protein Data Bank (PDB, http://www.rcsb.org), with ∼90% of its entries resulting from this method. Despite the rapid development in this field, there is still a major obstacle that limits crystallography, namely the production of high-quality crystals. Crystallization is an unpredictable process since it is mainly based on a random search for conditions that might lead to the formation of crystals (Drenth, 2007 ▸). The growth of high-quality crystals is hampered by the fact that proteins exhibit only a small number of crystal contacts and are held together by weak noncovalent interactions (Rupp, 2009 ▸). The free crystallization energy ΔG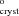 depends on both enthalpic (ΔH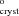) and entropic (TΔS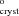) terms, and can be described as follows (Derewenda & Vekilov, 2006 ▸), 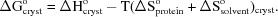 Owing to the small number and poor strength of intermolecular crystal contacts, the enthalpic contribution ΔH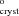 becomes, at best, only moderately negative. In addition, the incorporation of molecules into a crystal lattice costs entropy due to the loss of the protein molecule’s degree of freedom, making the term ΔS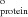 unfavorably negative. Therefore, the main driving force for crystallization is the significant increase of the solvent entropy ΔS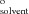, due to the release of solvent molecules from the hydration shell upon the formation of protein–protein contacts (Vekilov, 2003 ▸).

The phase behavior of proteins is very complex and under certain conditions proteins tend to form clusters that can separate from aqueous solution into a dense liquid phase, leading to a liquid–liquid phase separation (LLPS) zone (Vekilov, 2010 ▸). It has been demonstrated that short-range forces between protein molecules and the formation of protein clusters can result in an LLPS (Stradner *et al.*, 2004 ▸). The formation of an LLPS is frequently observed in protein crystallization and is usually found at high protein concentrations and low precipitant concentrations, and its formation can, in turn, result in the nucleation of protein crystals (Maes *et al.*, 2015 ▸). It was shown that multivalent cations (*e.g.* Y^3+^, Cd^2+^, Zn^2+^) can modulate the electrostatic interactions between acidic protein molecules (*e.g.* β-lactoglobulin, human serum, bovine serum albumin), leading to the controlled formation of an LLPS and resulting in cocrystallization of the protein molecules with these ions (Zhang *et al.*, 2010 ▸, 2011 ▸, 2012 ▸, 2014 ▸; Grimaldo *et al.*, 2015 ▸). Experiments revealed that the critical temperature for LLPS formation is drastically lowered by the addition of Y^3+^ and the results implied that the cation-induced LLPS is an entropy-driven process owing to the release of hydration solvent molecules (Matsarskaia *et al.*, 2016 ▸).

We reported recently on the successful usage of a multivalent anion, namely the Anderson–Evans-type polyoxotungstate hexatungstotellurate(VI), [TeW_6_O_24_]^6−^ (TEW) (see Fig. S1 in the supporting information), as a crystallization additive, leading to the crystallization of mushroom tyrosinase from *Agaricus bis­porus* (*ab*PPO4), aurone synthase from *Coreopsis grandiflora* (*cg*AUS1) and the model protein hen egg-white lysozyme (HEWL) (Mauracher *et al.*, 2014*a* ▸,*b*; Molitor *et al.*, 2015*a* ▸, 2016 ▸ *a,b*; Bijelic *et al.*, 2015 ▸). The protein crystals of the three proteins that were cocrystallized with TEW were obtained either within (*cg*AUS1 and HEWL) or very close to (*ab*PPO4) the LLPS zone. During the crystallization of these proteins, a series of other polyoxometalates (POMs), namely decavanadate [V_10_O_28_]^6−^, the Wells–Dawson anion [P_2_W_18_O_62_]^6−^ and the Preyssler anion [NaP_5_W_30_O_110_]^14−^, were also screened for their suitability as crystallization agents but they failed to crystallize our target proteins, especially *ab*PPO4. Thus, TEW was the most suitable additive among the tested POMs, as it was the only structure to produce diffracting crystals of our target proteins. The advantages of TEW over other POM archetypes were described recently (Bijelic & Rompel, 2015 ▸, 2017 ▸). Analysis of the *ab*PPO4–TEW structure revealed that the inorganic cluster was crucially involved in the crystal packing, inducing the unexpected cocrystallization of both the active and latent form of this enzyme in a single crystal (Mauracher *et al.*, 2014*a* ▸,*b*). A similar behavior of TEW was observed during cocrystallization with HEWL, resulting in a new crystal form and thus confirming the suitability of TEW as a crystallization additive (Bijelic *et al.*, 2015 ▸). In the case of *cg*AUS1, TEW was even able to dramatically improve the crystal quality (*i.e.* an increase in resolution by up to ∼1.0 Å) in comparison to the crystal forms grown in its absence (Molitor *et al.*, 2015*a* ▸, 2016 ▸ *a,b*).

We therefore report on the detailed investigation of *cg*AUS1 structures obtained in the presence and absence of TEW in order to elucidate the mode of action of TEW. Furthermore, we describe the contribution of TEW to the crystallization entropy *via* determination of the solvent-accessible surface area (ASA) and compare all three protein–TEW structures to demonstrate the particular aptitude of TEW as a powerful crystallization tool.

## 2. Experimental   

### 2.1. Purification, crystallization, and structure elucidation of *ab*PPO4, HEWL, and *cg*AUS1   

The purification, crystallization, and structure determination of *ab*PPO4, HEWL, and *cg*AUS1 have been described previously (Mauracher *et al.*, 2014*a* ▸,*b*; Molitor *et al.*, 2015*a* ▸,*b* ▸, 2016*a* ▸,*b* ▸; Bijelic *et al.*, 2015 ▸).

### 2.2. Crystal contact analysis   

Initially, the crystal contacts of all the crystal forms of *cg*AUS1 were analyzed with *PISA* (Krissinel & Henrick, 2007 ▸) by computing the total number of crystal contacts, inclusive of their respective areas, and number and kind of participating amino acids. For the contact areas of TEW-containing crystal structures, the protein–protein and protein–TEW contacts were first merged and the volumes of the overlapping residues were then subtracted to obtain the area of the protein–TEW–protein contacts. In addition, the crystal contacts were visualized with *PyMOL* (Schrödinger, LLC; http://www.pymol.org) by depicting all the residues of each monomer within a radius of 4.0 Å to obtain a clearer view of all the contacts and possible side-chain interactions.

### 2.3. Calculation of ΔASA   

**Figure 1:**
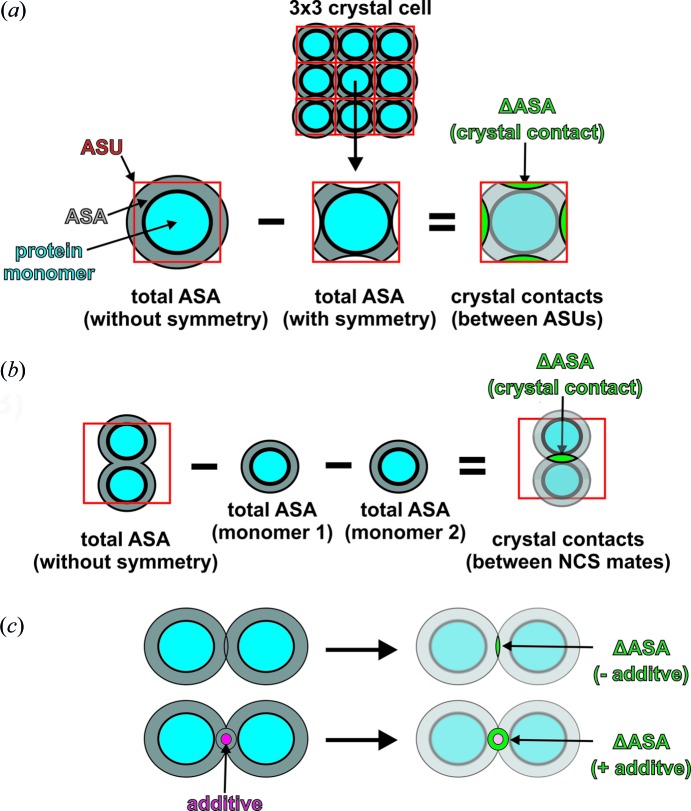
Schematic overview of the ASA calculations. (*a*) Calculation of the ΔASA(ASU) of an ASU containing one single monomer according to equation (1). ΔASA(ASU) was obtained by subtracting the total overall ASA of the ASU considering the interactions between symmetry mates from different ASUs [ASA(+symmetry), ASU–ASU interactions are indicated by the 3 × 3 crystal cell, middle] from the total overall ASA of the ASU excluding symmetry-mate interactions [ASA(−symmetry), left]. The resulting crystal contacts, ΔASA(ASU), are indicated by the green areas (right). Additives have been omitted for clarity. (*b*) Calculation of the ΔASA within the ASU containing two monomers. ΔASA(within ASU) was calculated using equation (2), namely by subtracting the total overall ASA of every monomer [ASA(monomer), middle] from the overall ASA excluding crystal packing but including interactions between NCS mates [ASA(−symmetry), left]. The resulting crystal contacts between NCS mates within the ASA are shown in green (right). Additives have been omitted for clarity and therefore the additive term, 
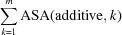
, of equation (2) has to be ignored for this graphic. To obtain the total ΔASA, ΔASA(total), for structures possessing more than one monomer in the ASU, the results of equations (1) (part *a*) and (2) (part *b*) had to be summed as shown in equation (3). (*c*) Possible impact of an additive on ΔASA as the crystal contact increases in the presence of the additive (bottom) compared to the same situation without the additive (top). The resulting difference areas are colored green.

The solvent-accessible surface area (ASA) was calculated with *AREAIMOL* (Saff & Kuijlaars, 1997 ▸; Lee & Richards, 1971 ▸) from the *CCP*4 suite (Winn *et al.*, 2011 ▸). A probe radius of 1.4 Å was used for each calculation. The program *AREAIMOL* determines the ΔASA values due to crystal packing by calculating the difference between the ASA of the asymmetric unit (ASU) without taking into account the crystal packing [ASA(−symmetry)] and the ASA of the ASU considering its contacts with the symmetry-related adjacent ASUs [ASA(+symmetry)] 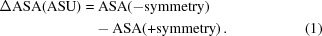
However, as several crystal structures contained more than one molecule within their ASU, the ΔASA value within the ASU (ΔASA within ASU) had additionally to be determined manually by calculating the difference area upon crystal contacts between noncrystallographic symmetry (NCS) mates 
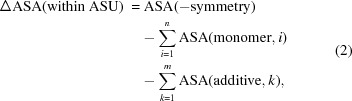
where ASA(−symmetry) is again the overall ASA of the entire ASU without considering the crystal packing (but including overlapping regions between monomers), ASA(monomer, *i*) is the total overall ASA (excluding contacts between symmetry mates from other ASUs) of monomer *i*, and ASA(additive, *k*) is the total overall ASA of additive *k* to include the contribution of the additive to ΔASA. The final and total ΔASA, ΔASA(total), was then obtained by summing the results from equations (1) and (2)

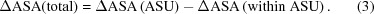
Fig. S2 (see supporting information) shows graphically the results of the *AREAIMOL* crystal contact determination for all three structures of *cg*AUS1. In order to analyze the impact of the different additives, we calculated the ΔASA values as described above for each investigated structure in the presence and absence of these additives by simply deleting the additive coordinates from the PDB file prior to the ASA calculation (Fig. 1 ▸ *c*). Additives were ranked by their impact on ΔASA, that is, whether ΔASA was significantly more reduced in their presence than in their absence. In cases of a significant reduction in ΔASA, the additives were assumed to have a crucial role in the crystal packing and thus in the solvent-based entropy term ΔS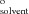, as the binding of some additives at the crystal contacts results in an enhanced release of solvent molecules.

## 3. Results and discussion   

**Figure 2:**
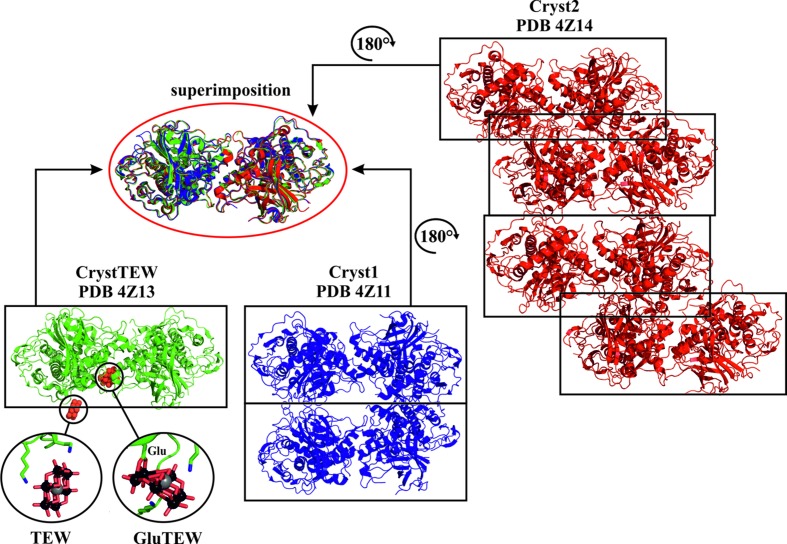
Comparison of the asymmetric units and the crystallographic dimer of the crystal forms CrystTEW, Cryst1, and Cryst2. The asymmetric units are indicated by boxes. CrystTEW is shown as a green cartoon, whereas Cryst1 is represented as a blue cartoon and Cryst2 as a red cartoon. Both TEW anions in CrystTEW (TEW and GluTEW) are shown as a cluster of red spheres within the cartoon but also in a ball-and-stick representation in the inset below (color code: tungsten black, tellurium gray, and oxygen red). The superimposition clearly indicates the presence of the same crystallographic dimer in each crystal form (r.m.s. deviation ∼0.45–0.72 Å).

Crystallization trials of *cg*AUS1 were accompanied by the occurrence of a high dense liquid phase (LLPS) and subsequent formation of undesired solid forms like amorphous precipitation, spherulites, needles, *etc*. (Molitor *et al.*, 2015*b* ▸). Two crystal forms of rather moderate quality were obtained, namely Cryst1 (PDB entry 4z11; Molitor *et al.*, 2016*b* ▸) and Cryst2 (PDB entry 4z14; Molitor *et al.*, 2016*b* ▸); however, their crystallization was not reproducible due to difficulties in controlling the nucleation and crystal growth. This problem was only resolved by applying TEW, leading to drastically improved crystallization that yielded crystals of much better quality (CrystTEW; Molitor *et al.*, 2016*a* ▸). Cryst1 and CrystTEW crystallized in the space group *P*12_1_1, whereas Cryst2 was obtained in the space group *P*1, containing different numbers of protein monomers per asymmetric unit (ASU), *viz.* Cryst1 had four monomers, Cryst2 had eight monomers, and CrystTEW had two monomers. The crystal lattices of all three crystal forms are composed of the identical crystallographic dimer, only differing in its orientation within the ASU, although the protein was shown to exist as a monomer in solution by size-exclusion chromatography and the interface exploring tool *PISA* (Molitor *et al.*, 2016*b* ▸) (Fig. 2 ▸). CrystTEW contains two TEW anions in its structure (see TEW and GluTEW in Fig. 2 ▸), with one of them being covalently bound to a glutamic acid (GluTEW) molecule, which is located at the interface of the crystallographic dimer. Both TEW anions mediate strong crystal contacts between a total of three crystallographic dimers (see Fig. S3 in the supporting information), whereby GluTEW mainly stabilizes the dimeric assembly (Molitor *et al.*, 2016*a* ▸).

**Figure 3:**
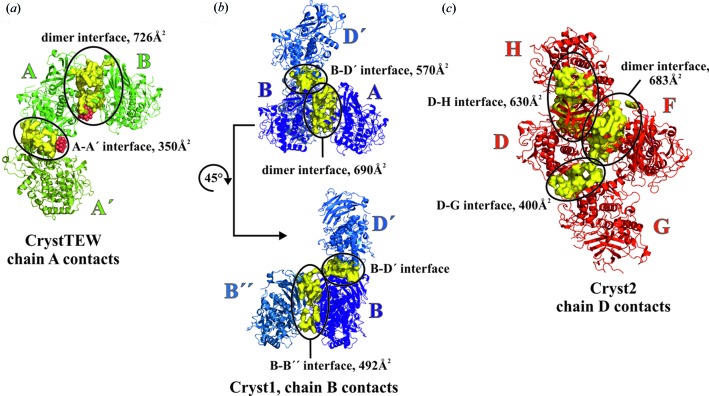
Comparison of crystal contacts of all *cg*AUS1 crystal forms. Only the strongest contacts of one monomer/chain of each crystal forms are illustrated, possessing a contact area of at least 400 Å [with the exception of CrystTEW (**A**), as there is no further large contact besides the dimeric interface]. (*a*) Crystal contacts of chain **A** of CrystTEW with adjacent monomers (both TEW molecules are illustrated as clusters of red spheres). (*b*) Crystal contacts of chain **B** of Cryst1 with adjacent monomers. (*c*) Crystal contacts of chain **D** with neighboring monomers. All chains are illustrated as cartoons. Monomers from adjacent ASUs are colored in different color shades and marked by single (′) and double (′′) primes, respectively. Crystal contacts are depicted as yellow surfaces and encircled to identify the respective contact.

The crystallographic dimer represents the strongest contact in all the crystal forms with regard to both contact area and number of participating amino acids, as calculated by *PISA* (Krissinel & Henrick, 2007 ▸) (see Table S1 in the supporting information). This dimer in CrystTEW covers an area of 726 Å^2^ and contains 50 interface-participating residues. The dimer interfaces of Cryst1 and Cryst2 are very similar, with average contact areas of 679 and 669 Å^2^, respectively, and about 44 contributing residues (Fig. 3 ▸). However, in CrystTEW, the GluTEW-mediated dimer interface is by far the strongest contact in the structure, followed by the second TEW-mediated contact, with an area of only 350 Å^2^ and 33 participating residues, whereas in the other two crystal forms, each monomer contains at least one or two further major contacts (with an area of at least 400 Å^2^) (Fig. 3 ▸, and Table S1 in the supporting information).

**Figure 4:**
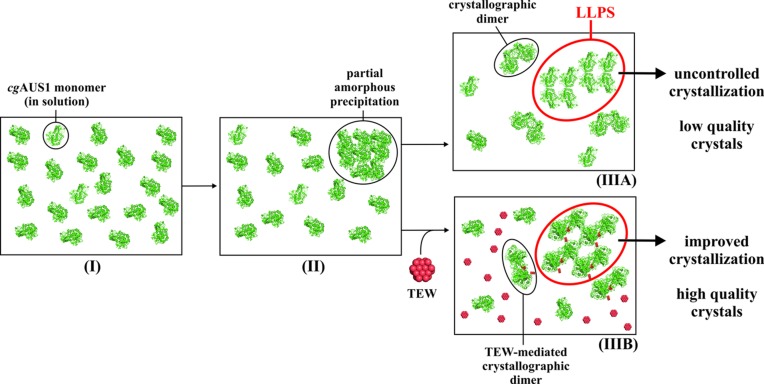
Schematic representation of the crystallization experiment of *cg*AUS1. (I) *cg*AUS1 after setting up the crystallization experiment; the protein exists as a monomer in solution. (II) Crystallization set-up after a few hours; partial precipitation occurs due to unspecific interactions between *cg*AUS1 monomers. (IIIA) Crystallization set-up after a longer period of time, the precipitate dissolves again under the formation of a high dense protein phase (LLPS) containing clusters of protein molecules (here the tetrameric arrangement of cgAUS1 is shown within the LLPS built of the crystallographic dimer). (IIIB) The same scenario as in (IIIA), but cocrystallized with TEW. As all crystal forms obtained are composed of the same crystallographic dimer, it is very likely that these dimers are crucial for the formation of the LLPS and thus the crystallization process. Nucleation and crystal growth was difficult to control within the LLPS zone in the absence of TEW (IIIA), which resulted in crystal forms of lower quality. However, in the presence of TEW (IIIB), both nucleation and crystal growth were dramatically improved, leading to a new crystal form of higher quality. *cg*AUS1 molecules are depicted as green cartoons, TEW is represented as a cluster of red spheres, and the LLPS is marked by a red circle.

Based on these results, it is very likely that the dimeric assembly plays a crucial role in protein cluster formation, which subsequently initiates the nucleation process. The GluTEW-mediated dimer interface seems to be clearly favored, as it exceeds other possible and maybe unspecific contacts and directs the LLPS-based crystallization process to a more ordered crystal of higher quality (Fig. 4 ▸). This could also explain the undesired outcomes of the crystallization trials in the absence of TEW since too unspecific interactions between crystallographic dimers lead to a less ordered assembly of protein molecules. A similar observation was made during TEW-mediated crystallization of HEWL, where TEW induced an unprecedented tetrameric arrangement of HEWL, leading to a TEW-directed crystal growth and finally to a new crystal form (Bijelic *et al.*, 2015 ▸). It should be noted that all crystallization conditions leading to the TEW-mediated crystallization of *ab*PPO4, HEWL, and *cg*AUS1, formed an LLPS also in the absence of TEW (but no crystals were obtained from this LLPS without TEW in the case of *ab*PPO4 and HEWL). Therefore, it is suggested that TEW should be tried, especially under conditions leading to an LLPS, as within this phase zone, the polyanion was shown to be at its most effective.

To identify further beneficial features of TEW on protein crystallization, we investigated its potential effects on the crystallization energy by calculating the solvent-accessible surface area (ASA) of all the crystal forms of *cg*AUS1. In this way, it is possible to compare the respective contributions of crystal packing with ΔS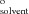 representing the driving force of crystallization. The ΔASA values were calculated with *AREAIMOL* (Saff & Kuijlaars, 1997 ▸; Lee & Richards, 1971 ▸) (Table 1 ▸). The results revealed that CrystTEW is the entropically most favored crystal form, as more solvent is released during its crystal packing than in Cryst1 and Cryst2. Fig. S4 (see supporting information) shows schematically the process of water release and the associated increase of the entropy upon TEW binding. However, the crystal packing of Cryst1 and Cryst2 is preferred when omitting both TEW molecules in CrystTEW (Table 1 ▸, values in parentheses), proving that the favored crystal packing is TEW-mediated. The ASA data also show that the average protein–TEW interactions contribute substantially, with a value of ∼690 Å^2^, to the overall ASA difference (∼24%). Remarkably, similar contributions were also found for the structures of *ab*PPO4 (∼22%) and HEWL (∼35%) (Table 1 ▸).

As HEWL is a standard protein in crystallization and thus abundant in the PDB, we compared the crystal form of HEWL–TEW (PDB entry 4phi; Bijelic *et al.*, 2015 ▸) with a series of reference HEWL structures (see Table S2 in the supporting information). The results indicate that TEW is clearly superior over commonly used additives (I^−^, NO_3_^−^, *etc*.) in terms of entropy, even when a large number accumulate at the protein–protein interface (*e.g.* one TEW molecule is able to compensate up to 13 I atoms). Only one group of additives exhibited similar or even slightly more favorable effects on the ASA of HEWL, namely Eu-containing dipicolinate (dpa) complexes (see Table S3 and Fig. S5 in the supporting information) (Pompidor *et al.*, 2010 ▸; Talon *et al.*, 2012 ▸). These complexes were used as phasing tools and are involved in intermolecular protein–protein contacts, not only *via* electrostatic, but also *via* hydro­phobic π–π stacking interactions with aromatic residues. However, only Eu complexes of significantly greater size than TEW led to the release of more water molecules, indicating that it is not only size that plays a crucial role in water release, but also the charge and shape of the molecule. Similar comparisons with *ab*PPO4 were not possible due to the lack of appropriate TEW-less reference structures.

The previously reported effect of multivalent cations (Y^3+^) on acidic proteins is similar to that of TEW on proteins bearing positively charged patches. According to the ΔASA values of the Y^3+^- and Zn^2+^-induced crystallization of β-lacto­globulin (PDB entries 4lzu, 4lzv, 3ph5 and 3ph6; Zhang *et al.*, 2011 ▸), the cations have a significantly more minor effect on solvent release than TEW in its structures (ΔASA contribution: TEW ≃ −340 Å^2^, Y^3+^ ≃ −41 Å^2^, and Zn^2+^ ≃ −3 Å^2^) (see Table S4 in the supporting information); however, it was shown by isothermal titration calorimetry that the entropy contribution of the Y^3+^–protein interactions is sufficient to induce a special phase behavior within protein solutions from which protein crystals can grow (Matsarskaia *et al.*, 2016 ▸). Therefore, it can be concluded that the influence of TEW on the total crystallization entropy is also significant and in some cases could be immense.

## 4. Conclusion   

In summary, our results demonstrate that the beneficial effects of TEW on protein crystallization within the LLPS is based on TEW–protein binding and protein–protein linking, which are both entropy-driven processes, owing to the release of solvent molecules from their hydration shells into the bulk water environment. This leads to a significant gain in the total crystallization entropy, making TEW a powerful crystallization agent.

## 5. Related literature   

References cited in the supporting information include: Artymiuk *et al.* (1982 ▸), Brinkmann *et al.* (2006 ▸), Majeed *et al.* (2003 ▸), Vaney *et al.* (2001 ▸), Walsh *et al.* (1998 ▸), Yamada *et al.* (2015 ▸) and Zander *et al.* (2016 ▸).

## Supplementary Material

Supplementary figures and tables.. DOI: 10.1107/S2052252517012349/lq5006sup1.pdf

## Figures and Tables

**Table 1:** ΔASA values of all TEW-containing crystal structures and of Cryst1 and Cryst2

	CrystTEW4z13	Cryst14z11	Cryst24z14	*ab*PPO44oua	HEWL4phi
Space group	*P*2_1_	*P*2_1_	*P*1	*C*2	*P*4_3_2_1_2
Additive	[TeW_6_O_24_]^6−^	MgCl_2_	MgCl_2_	[TeW_6_O_24_]^6−^	[TeW_6_O_24_]^6−^
No. of additives within interfaces	2 of 2	—†	—†	2 of 2	8 of 8
Reference	Molitor *et al.* (2016*a* ▸,*b* ▸)	Molitor *et al.* (2016*a* ▸,*b* ▸)	Molitor *et al.* (2016*a* ▸,*b* ▸)	Mauracher *et al.* (2014*b* ▸)	Bijelic *et al.* (2015 ▸)
Crystal contacts of ASU‡					
ΔASA(ASU) (Å^2^)	−3383.8 (−3010.9)§	−6022.1 —†	−10449.1 —†	−4413.2 (−3901.3)§	−6238.5 (−4344.7)§
Per monomer (Å^2^)	−1691.9 (−1505.5)§	−1505.5 —†	−1306.1 —†	−2206.6 (−1950.7)§	−1559.6 (−1086.2)§
Crystal contacts within ASU¶					
ΔASA(within ASU) (Å^2^)	−2414.3 (−1436.4)§	−3971.8 —†	−9511.8 —†	−1901.5 (−1037.3)§	−7356.9 (−4446.6)§
Per monomer (Å^2^)	−1207.2 (−718.2)§	−993.0 —†	−1189.0 —†	−950.8 (−518.7)§	−1839.2 (−1111.7)§
ΔASA_total_ ††					
Per monomer (Å^2^)	−2899.1(−2154.6)§	−2498.5—†	−2495.1—†	−3157.4(−2469.3)§	−3398.9(−2197.8)§
Additive contribution to ΔASA per molecule (Å^2^)‡‡	−744.5	—†	—†	−688.1	−600.6

† This value was omitted since MgCl_2_ did not exhibit a significant impact on the ΔASA.

‡ Crystal contacts of ASU [= ΔASA(ASU)] describes the contacts obtained by equation (1), that is, contacts between monomers originating from different ASUs (see Fig. 1 ▸ *a*).

§ The values in parentheses represent area differences without taking into account the TEW molecules by deleting them from the PDB file in order to analyse their impact on the crystal contacts.

¶ Crystal contacts within ASU [= ΔASA(within ASU)] describes the contacts obtained by equation (2), that is, contacts between NCS mates (only necessary for ASUs containing more than one monomer) (Fig. 1 ▸ *b*).

†† ΔASA_total_ = ΔASA(ASU) + ΔASA(within ASU), as described in equation (3).

‡‡ This value was obtained by simply subtracting the ΔASA_total_ per monomer value for the system ignoring TEW (value in parentheses) from that including TEW.
