# Supplementary material for: The potential of hexatungstotellurate(VI) to induce a significant entropic gain during protein crystallization
Source: IUCrJ. 2017 Oct 27;4(Pt 6):734–40. doi: 10.1107/S2052252517012349 (PMC5668858; doi:10.1107/S2052252517012349)
Supplement: Supplementary file 1 [file m-04-00734-sup1.pdf]

IUCrJ

Volume 4 (2017)

Supporting information for article:

The potential of hexatungstotellurate(VI) to induce a significant entropic gain during protein crystallization

Christian Molitor, Aleksandar Bijelic and Annette Rompel

S1. Figures

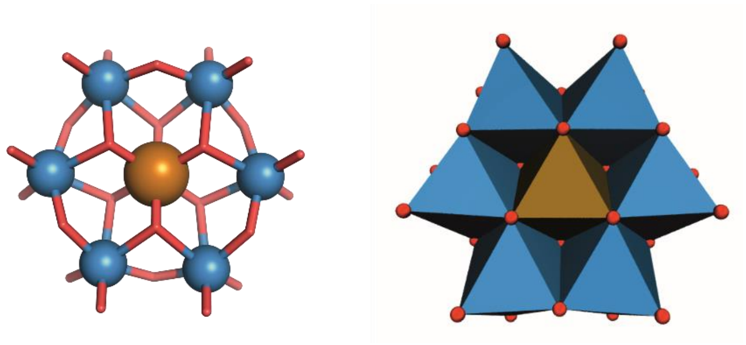

Figure S1 Structures of $[TeW_6O_{24}]^{6-}$. TEW is shown in ball and stick (left) and polyhedra (right) representation. Color code: tungsten, cyan; tellurium; brown; oxygen, red.

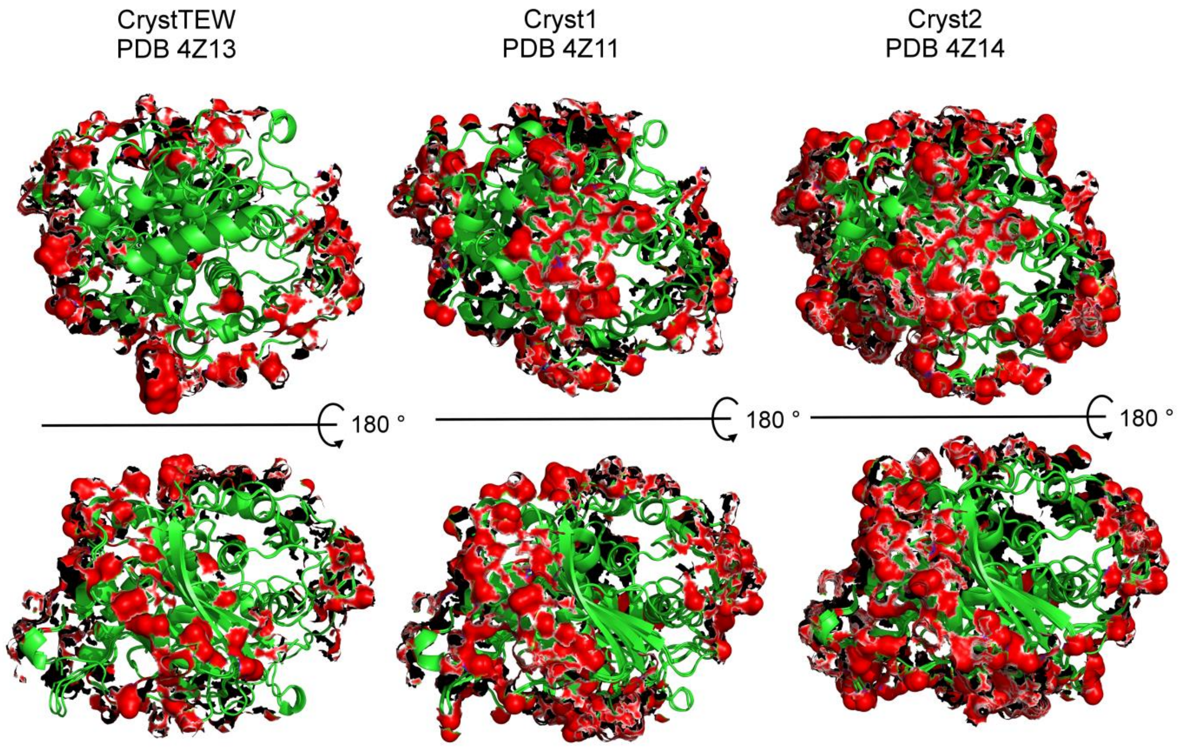

Figure S2 Graphic representation of the ΔASA calculation results for all three structures of *cg*AUS1. The crystal contacts (ΔASA) of the respective crystal form were determined by AREAIMOL. The protein structures are shown as green cartoons, whereas the calculated crystal contacts are illustrated as red surfaces.

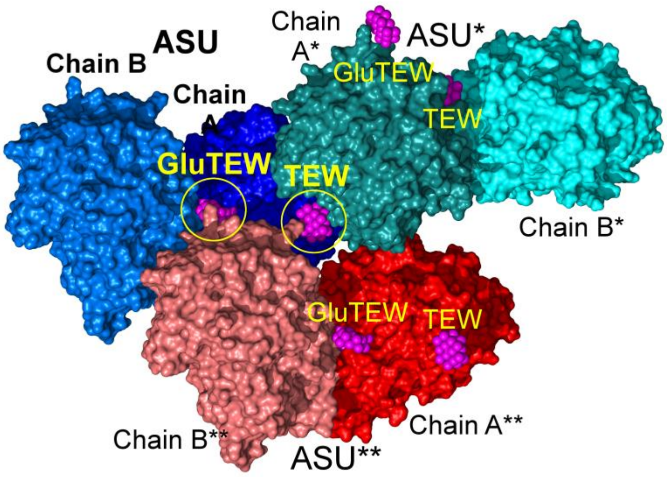

Figure S3 Impact of TEW on the crystal packing of *cg*AUS1. Both TEW anions (GluTEW is the TEW ion which is covalently bound to a glutamic acid) strongly influence the crystal packing of *cg*AUS1 in CrystTEW, as both mediate new crystal contacts between symmetry related crystallographic dimers.

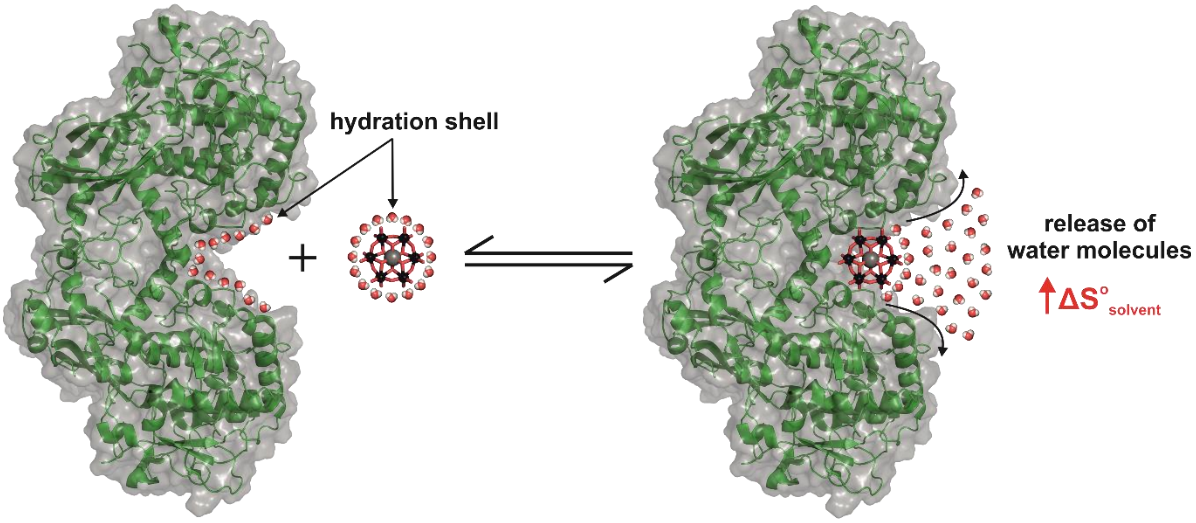

Figure S4 Schematic illustration of the water release event upon TEW binding. Before the binding event both, TEW (or the additive in general) and the protein, are hydrated, that is, surrounded by a hydration shell (indicated by water molecules shown as small spheres with the red sphere being the oxygen atom and the white spheres being the hydrogen atoms). Upon TEW-protein binding, the release of water molecules from their hydration shells causes a gain in solvent entropy. The protein (*cg*AUS1) is shown as a green cartoon, which is surrounded by a grey transparent surface to illustrate the binding site of TEW and the crystal contact. TEW is illustrated in ball and stick representation with the following color code: tellurium, grey; tungsten, black; oxygen, red. The figure is inspired by Figure 3 from (Matsarskaia *et al.*, 2016).

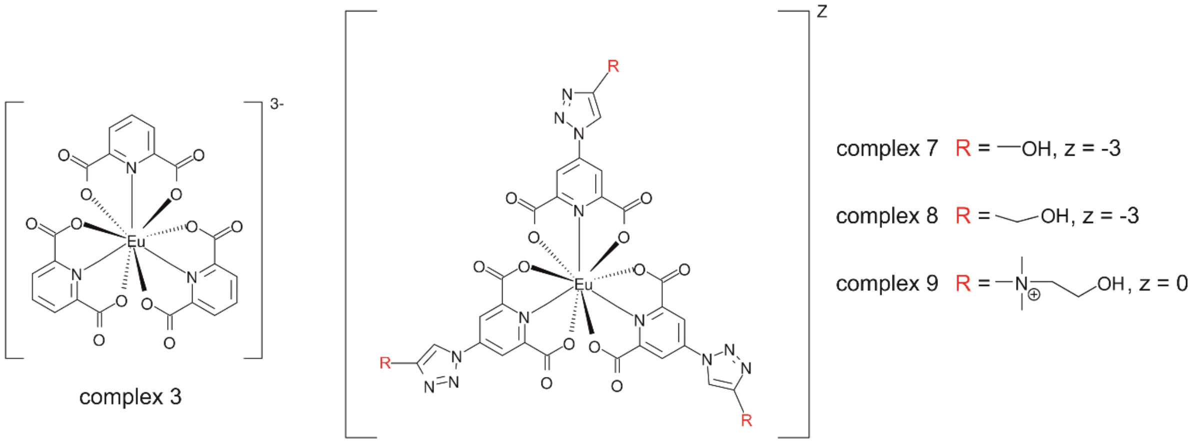

Figure S5 Structure of the Eu containing dipicolinate complexes.

S2. Tables

Table S1 Crystal contacts of all crystal forms of *cg*AUS1 calculated by PISA.

Crystal form	Contact (by chain)	Contact area (PISA)[a]	No. of contact residues (PISA)
CrystTEW	A-B[b]	726 Å^2	50
	A-A	350 Å^2	33
Cryst1	A-B[b]	690 Å^2	44
	C-D[b]	668 Å^2	43
	B-D	570 Å^2	41
	B-B	492 Å^2	31
	D-D	487 Å^2	30
	A-C	419 Å^2	38
Cryst2	C-E[b]	701 Å^2	44
	D-F[b]	683 Å^2	46
	B-H[b]	665 Å^2	41
	A-G[b]	629 Å^2	40
	D-H	630 Å^2	51
	C-G	572 Å^2	47
	E-E	490 Å^2	32
	B-B	483 Å^2	30
	F-F	471 Å^2	29
	A-A	466 Å^2	27

[a] Note that only the largest contacts (> 400 Å^2) were considered, however, with exemption of CrysTEW where a smaller contact is shown due to the lack of further large/strong contacts.

[b] This crystal contact represents a crystallographic dimer building up each crystal form.

Table S2 **ΔASA values of reference HEWL structures. Representatives chosen from every reported space group.**

	HEWL PDB 4LZT	HEWL PDB 1B2K	HEWL PDB 1PS5	HEWL PDB 2FBB	HEWL PDB 1AKI	HEWL PDB 4WM6	HEWL PDB 5EBH
**Space group**	P1	$P12_11$	C121	$P6_122$	$P2_12_12_1$	$P4_3$	$P4_32_12$
**Additive**	NO_3^-	I^-	SO_4^{2-}	NO_3^-	-	-	-
**No. of additives within interfaces**	5 of 6	9 of 17	1 of 2	9 of 16	-	-	-
**Reference**	[(Walsh *et al.*, 1998)]	[(Vaney *et al.*, 2001)]	[(Majeed *et al.*, 2003)]	[(Brinkmann *et al.*, 2006)]	[(Artymiuk *et al.*, 1982)]	[(Yamada *et al.*, 2015)]	[(Zander *et al.*, 2016)]
**Crystal contacts of ASU**[c]							
ΔASA(ASU) [Å^2]	-3256.7 (-3039.9)[a]	-5754.6 (-5152.7)[a]	-2434.8 (-2402.4)[a]	-2442,4 (-2142.3)[a]	-2315.3 -	-7310.0 -	-1842.3 -
Per monomer [Å^2]	-3256.7 (-3039.9)[a]	-2877.3 (-2576.4)[a]	-2434.8 (-2402.4)[a]	-2442.4 (-2142.3)[a]	-2315.3 -	-1827.5 -	-1842.3 -
**Crystal contacts within ASU**[d]							
ΔASA(within ASU) [Å^2]		-1663.5 (-552.6)[a]	-[b]	-[b]	-[b]	-1389.7	-[b]
Per monomer [Å^2]		-831.8 (-276.3)[a]	-[b]	-[b]	-[b]	-347.4	-[b]
**ΔASA_{total}**[e]							
Per monomer [Å^2]	-3256.7 (-3039.9)[a]	-3709.1 (-2852.7)[a]	-2434.8 (-2402.4)[a]	-2442.4 (-2142.3)[a]	-2315.3 -	-2174.9 -	-1842.3 -
**Additive contribution to ΔASA per molecule [Å^2]**[f]	-43.4	-95.2	-16.2	-33.3	-[b]	-[b]	-[b]

[a] The values in parentheses represent area differences without taking into account the additives by deleting them from the pdb file in order to analyse their impact on the crystal contacts. [b] ASU contains only one monomer. [c] Crystal contacts of ASU (=ΔASA(ASU)) describes the contacts obtained by equation (1), that is, contacts between monomers originating from different ASUs (see Figure 1A). [d] Crystal contacts within ASU (= ΔASA(within ASU)) describes the contacts obtained by equation (2), that is, contacts between NCS mates (only necessary for ASUs containing more than one monomer) (Figure 1B). [e] ΔASA_{total} = ΔASA(ASU) + ΔASA(within ASU) as described in equation (3). [f] This value was obtained by simply subtracting the ΔASA_{total} per monomer value for the system ignoring the additive (value in parentheses) from that including the additive.

Please note that Table S2 is just a selection of the examined HEWL structures. Only one structure per space group is provided in order to show ΔASA values for all reported space groups. Commonly applied additives in HEWL structures are mostly different anions (e.g. NO_3^-, I^-, Br^-, SCN^-, Cl^-, etc.), however, they do not significantly contribute to ΔASA and were therefore omitted.

Table S3 Impact of different Eu(dpa)$_3$ complexes on ΔASA of HEWL due to crystal packing

	HEWL PDB 2PC2	HEWL PDB 4BAD	HEWL PDB 4BAF	HEWL PDB 4BAP
**Space group**	C2	$P4_32_12$	$P4_32_12$	$P4_32_12$
**Additive**	Eu(dpa)$_3$ complex 3[c]	Eu(dpa)$_3$ Complex 7[c]	Eu(dpa)$_3$ complex 8[c]	Eu(dpa)$_3$ complex 9[c]
**No. of additives within interfaces**	4 of 5	1 of 1	1 of 1	1 of 1
**Reference**	[(Talon *et al.*, 2012)]	[(Talon *et al.*, 2012)]	[(Talon *et al.*, 2012)]	[(Talon *et al.*, 2012)]
**Crystal contacts of ASU[d]**				
ΔASA(ASU) [Å^2]	-4259.3 (-2202.3)[a]	-3074.1 (-2361.4)[a]	-2991.1 (-2179.5)[a]	-3287.9 (-2301.2)[a]
Per monomer [Å^2]	-4259.3 (-2202.3)[a]	-3074.1 (-2361.4)[a]	-2991.1 (-2179.5)[a]	-3287.9 (-2301.2)[a]
**Crystal contacts within ASU[d]**				
ΔASA(within ASU) [Å^2]	-[b]	-[b]	-[b]	-[b]
Per monomer [Å^2]	-[b]	-[b]	-[b]	-[b]
**ΔASA$_{total}$[e]**				
Per monomer [Å^2]	-4259.3 (-2202.3)[a]	-3074.1 (-2361.4)[a]	-2991.1 (-2179.5)[a]	-3287,9 (-2301.2)[a]
**Additive contribution to ΔASA per molecule [Å^2][f]**	-533.5	-712.7	-811.6	-986.7

[a] The values in parentheses represent area differences without taking into account the additives by deleting them from the pdb file in order to analyse their impact on the crystal contacts. [b] ASU contains only one monomer. [c] For structure see Figure S3. [d] Crystal contacts of ASU (=ΔASA(ASU)) describes the contacts obtained by equation (1), that is, contacts between monomers originating from different ASUs (see Figure 1A). [e] Crystal contacts within ASU (= ΔASA(within ASU)) describes the contacts obtained by equation (2), that is, contacts between NCS mates (only necessary for ASUs containing more than one monomer) (Figure 1B). [f] ΔASA$_{total}$ = ΔASA(ASU) + ΔASA(within ASU) as described in equation (3). [g] This value was obtained by simply subtracting the ΔASA$_{total}$ per monomer value for the system ignoring the additive (value in parentheses) from that including the additive. [e] ΔASA$_{total}$ = ΔASA(ASU) + ΔASA(within ASU) as described in equation (3).

Please note that during the investigation of HEWL structures also other additives were found and analysed, however, they did not significantly contribute to ΔASA and were therefore omitted.

Table S4 **ΔASA values of β-lactoglobulin (BLG) in presence of Y^{3+} and Zn^{2+}.**

	BLG PDB 4LZU	BLG PDB 4LZV	BLG PDB 3PH5	BLG PDB 3PH6
**Space group**	$P3_221$	$P3_221$	$P2_12_12_1$	$P2_12_12_1$
**Additive**	Zn^{2+}	Zn^{2+}	Y^{3+}	Y^{3+}
**No. of additives within interfaces**	1 of 2	1 of 3	4 of 4	4 of 4
**Reference**	- [a]	- [a]	[(Zhang *et al.*, 2011)]	[(Zhang *et al.*, 2011)]
**Crystal contacts of ASU**[c]				
ΔASA(ASU) [$Å^2$]	-1580.8 (-1577.8)[b]	-2022.9 (-2018.0)[b]	-1696.2 (-1676.2)[b]	-1781.0 (-1760.0)[b]
Per monomer [$Å^2$]	-1580.8 (-1577.8)[b]	-2022.9 (-2018.0)[b]	-1696.2 (-1676.2)[b]	-890.5 (-880.0)[b]
**Crystal contacts within ASU**[d]				
ΔASA(within ASU) [$Å^2$]	-[c]	-[c]	-1087.5 (-942.3)	-1109.9 (-965.0)
Per monomer [$Å^2$]	-[c]	-[c]	-543.8 (-471.2)	-555.0 (-482.5)
**ΔASA_{total}**[e]				
Per monomer [$Å^2$]	-1580.8 (-1577.8)[b]	-2022.9 (-2018.0)[b]	-1391.9 (-1309.3)[b]	-1445.5 (-1362.5)[b]
**Additive contribution to ΔASA per molecule [$Å^2$]**[f]	-3.0	-2.2	-41.3	-41.5

[a] PDB entry with no associated publication. [b] The values in parentheses represent area differences without taking into account the additives by deleting them from the pdb file in order to analyse their impact on the crystal contacts. [c] ASU contains only one monomer. [d] Crystal contacts of ASU (=ΔASA(ASU)) describes the contacts obtained by equation (1), that is, contacts between monomers originating from different ASUs (see Figure 1A). [e] Crystal contacts within ASU (= ΔASA(within ASU)) describes the contacts obtained by equation (2), that is, contacts between NCS mates (only necessary for ASUs containing more than one monomer) (Figure 1B). [f] ΔASA_{total} = ΔASA(ASU) + ΔASA(within ASU) as described in equation (3). [g] This value was obtained by simply subtracting the ΔASA_{total} per monomer value for the system ignoring the additive (value in parentheses) from that including the additive.

S3. References

Artymiuk, P. J., Blake, C. C. F., Rice, D. W. & Wilson, K. S. (1982). *Acta Crystallogr. B*. **38**, 778–783.

Brinkmann, C., Weiss, M. S. & Weckert, E. (2006). *Acta Crystallogr. D Biol. Crystallogr.* **62**, 349–355.

Majeed, S., Ofek, G., Belachew, A., Huang, C., Zhou, T. & Kwong, P. D. (2003). *Structure*. **11**, 1061–1070.

Matsarskaia, O., Braun, M. K., Roosen-Runge, F., Wolf, M., Zhang, F., Roth, R. & Schreiber, F. (2016). *J. Phys. Chem. B*. **120**, 7731–7736.

Talon, R., Nauton, L., Canet, J.-L., Kahn, R., Girard, E. & Gautier, A. (2012). *Chem. Commun.* **48**, 11886–11888.

Vaney, M. C., Broutin, I., Retailleau, P., Douangamath, A., Lafont, S., Hamiaux, C., Prangé, T., Ducruix, A. & Riès-Kautt, M. (2001). *Acta Crystallogr. D Biol. Crystallogr.* **57**, 929–940.

Walsh, M. A., Schneider, T. R., Sieker, L. C., Dauter, Z., Lamzin, V. S. & Wilson, K. S. (1998). *Acta Crystallogr. D Biol. Crystallogr.* **54**, 522–546.

Yamada, H., Nagae, T. & Watanabe, N. (2015). *Acta Crystallogr. D Biol. Crystallogr.* **71**, 742–753.

Zander, U., Hoffmann, G., Cornaciu, I., Marquette, J.-P., Papp, G., Landret, C., Seroul, G., Sinoir, J., Röwer, M., Felisaz, F., Rodriguez-Puente, S., Mariaule, V., Murphy, P., Mathieu, M., Cipriani, F. & Márquez, J. A. (2016). *Acta Crystallogr. Sect. Struct. Biol.* **72**, 454–466.

Zhang, F., Zocher, G., Sauter, A., Stehle, T. & Schreiber, F. (2011). *J. Appl. Crystallogr.* **44**, 755–762.
